# Long-Term Outcomes of Hyperadrenergic Orthostatic Hypotension

**DOI:** 10.21203/rs.3.rs-3318273/v1

**Published:** 2023-09-11

**Authors:** Robert Alexander Castro, Patricio Millar Vernetti, Italo Biaggioni, Satish R. Raj, Horacio Kaufmann, Cyndya A. Shibao

**Affiliations:** Vanderbilt University Medical Center; NYU Langone Health; Vanderbilt University Medical Center; Libin Cardiovascular Institute of Alberta; NYU Langone Medical Center: NYU Langone Health; Vanderbilt University Medical Center

**Keywords:** hyperadrenergic, orthostatic hypotension, norepinephrine, all-cause mortality, phenoconversion

## Abstract

**Purpose:**

Hyperadrenergic orthostatic hypotension is a subtype of orthostatic hypotension associated with elevated norepinephrine levels upon standing. Our previous study found that this subtype is characterized by less severe autonomic impairment compared to orthostatic hypotension with normal or low norepinephrine levels. However, long-term outcomes have not been determined. Thus, the purpose of this study was to evaluate the all-cause mortality and phenoconversion over 7 years.

**Methods:**

In this prospective observational study, 92 patients with orthostatic hypotension were recruited from the Vanderbilt Autonomic Dysfunction Center. 34 patients with upright norepinephrine levels above 600 pg/mL were included in the hyperadrenergic cohort and 58 composed the orthostatic hypotension cohort. Both cohorts were followed for 7 years while assessing all-cause mortality and phenoconversion to neurodegenerative autonomic disorders.

**Results:**

Hyperadrenergic patients showed an exaggerated orthostatic increase in norepinephrine to 938 ± 305 pg/mL upon head up tilt despite presenting with impaired autonomic reflexes. The 7-year mortality rate was 35% in the hyperadrenergic cohort compared to 22% in orthostatic hypotension (p = 0.01). The hyperadrenergic cohort had a greater phenoconversion rate to multiple system atrophy (p = 0.04), whereas the orthostatic hypotension cohort had greater phenoconversion to Parkinson’s disease and dementia with Lewy bodies.

**Conclusions:**

Despite having less severe autonomic impairment, our data suggests that hyperadrenergic orthostatic hypotension has worse clinical outcomes than neurogenic orthostatic hypotension. Patients with hyperadrenergic orthostatic hypotension require careful monitoring, given that this condition may be associated with negative outcomes.

## Introduction

Orthostatic hypotension (OH) is characterized by a decrease of more than 20 mmHg in systolic blood pressure (SBP) or 10 mmHg in diastolic blood pressure (DBP) within three minutes of standing or head up tilt. This condition results from impaired cardiovascular autonomic reflexes, particularly blunted sympathetic activation as detected by the absence of an increase in norepinephrine levels on standing. In addition, norepinephrine values while supine can inform about the level of the lesion within the autonomic reflex arch. Low norepinephrine levels occur with peripheral or post-ganglionic sympathetic denervation, whereas patients with central forms of autonomic failure, i.e., multiple system atrophy (MSA) present with normal or even high norepinephrine levels indicating a pre-ganglionic lesion.

Nevertheless, we previously reported a novel, distinct sub-phenotype of OH associated with an exaggerated increase in norepinephrine levels upon standing despite evidence of impaired sympathetic vasoconstriction, which we named hyperadrenergic orthostatic hypotension (hyperOH) [1]. Although hyperOH patients had less severe autonomic impairment compared to OH without this trait [1], the long-term outcomes and all-cause mortality have yet to be determined.

Further, given that normal or increased norepinephrine is often associated with phenoconversion to neurodegenerative autonomic disorders like MSA, we hypothesized that hyperOH patients could represent an early-stage autonomic neuropathy with a distinct pathophysiology and prognosis. Thus, the purpose of this prospective observational study was to determine the 7-year incidence of all-cause mortality and phenoconversion to neurodegenerative autonomic disorders for patients with hyperOH.

## Methods

This work was approved by the Vanderbilt University Institutional Review Board and has been performed in accordance with the ethical standards laid down in the 1964 Declaration of Helsinki and its later amendments.

### Patient Population

This is a prospective, observational study composed by two longitudinal cohorts conducted in a single institution, Vanderbilt University Medical Center. The initial cohort reported by Mar et al. was composed of 84 OH and hyperOH patients who were enrolled between August 2007 and May 2013 [1]. The initial Natural History Study of Pure Autonomic Failure cohort (clinicaltrials.gov NCT01799915) consisted of patients with OH and low or normal norepinephrine levels, i.e., pure autonomic failure (PAF). Using these two longitudinal cohorts, our combined cohort recruited 92 patients with OH and hyperOH.

All patients performed autonomic function testing (AFTs) that confirmed OH diagnosis, defined by the absence of a phase IV overshoot in blood pressure during the Valsalva Maneuver along with a significant orthostatic decrease in blood pressure. All patients underwent head-up tilt testing with supine and upright blood draws to assess plasma norepinephrine levels and to group participants into hyperOH or OH based on established criteria (upright plasma norepinephrine > 600 pg/mL) [1]. During tilt testing, all patients experienced an orthostatic reduction in SBP greater than 20 mmHg or an orthostatic reduction in DBP greater than 10 mmHg without a significant increase in heart rate. Patients with a square-root wave pattern during the Valsalva Maneuver were excluded from the study [1].

### Initial Testing

Initial AFTs included respiratory sinus arrythmia, hyperventilation, and Valsalva Maneuver during continuous monitoring of beat-to-beat blood pressure, heart rate (HR), heart rhythm, and respirations. NexFin (BMEYE, Amsterdam, The Netherlands) devices were used to record continuous blood pressure by the finger volume clamp method [1]. The Ivy monitor (Vital-Guard 450C, Ivy Biomedical Systems, Connecticut, USA) was used to record HR and heart rhythm via 5-lead electrocardiogram (ECG), and blood pressure via an automated brachial cuff. Data were collected using the WINDAQ (DI720, DATAQ Instruments, Ohio, USA) data acquisition system.

Magnitude of orthostatic decrease in blood pressure, Valsalva ratio, pressure recovery time (PRT), and the ratio of orthostatic change in HR to orthostatic change in SBP (ΔHR/ΔSBP ratio) were used to assess severity of autonomic impairment during initial reports [3].

### Norepinephrine Assessment

During the tilt test, patients first laid supine for 10 minutes to obtain a steady baseline state. Patients were then safely strapped to the tilt table and tilted to 75 degrees for up to ten minutes, or as long as they were able to remain upright. Plasma norepinephrine levels were assessed by blood draw when the patients were in the supine position and at 75-degree head up tilt. Samples were centrifuged at 3500 rpms for 10 minutes at 4 degrees Celsius and plasma were transferred into tubes with reduced 6% glutathione and placed in a −80 degree freezer until they were analyzed using high-performance liquid chromatography.

### Prospective Study Design

In accordance with the goals of this study, patients were followed for more than 7 years after initial report. We reviewed patients’ charts to collect demographic data for age, sex, height, weight, BMI, and medication use. Our team assessed available health records and contacted patients by phone to obtain yearly outcome data. Clinical and laboratory evaluations that were assessed included all-cause mortality, changes in diagnosis from PAF to MSA, Parkinson’s disease (PD), and dementia with Lewy bodies (DLB), and causes of death. At each yearly follow-up, the patients’ diagnoses were re-evaluated to evaluate whether they had developed clinical evidence of MSA, PD, or DLB, or if they retained an OH phenotype. The causes of death and changes in diagnosis were ascertained by attending physicians in accordance with current consensus criteria [2, 4].

### Statistical Analyses

All data were collected in REDCap, a secure, web-based application designed to support data capture. Prism 9 (GraphPad Software, Massachusetts, USA) software was used to analyze the data collected throughout the study. Student’s t tests were used to assess significant differences in demographics and clinical characteristics. Chi-squared tests were used to determine significant differences in outcomes over 7 years.

## Results

### Study Population

The hyperOH sample included 34 participants, made up of 19 men and 15 women. Their ages ranged from 43 to 87 with a mean of 70 ± 10 years (mean ± standard deviation), and a BMI of 24.3 ± 3.9 kg/m^2^. The medications used among this group included beta blockers, benzodiazepines, serotonin-norepinephrine reuptake inhibitors (SNRIs), selective serotonin reuptake inhibitors (SSRIs), opioid analgesics, and alpha-adrenergic agonists [1]. After 7 years, 12 patients were lost to follow-up, leaving 22 patients (65% of initial sample) with complete follow-up.

The OH cohort was made up of 58 patients (37 males, 21 females). Their age distribution was 70 ± 9 years and their BMI was 27.9 ± 4.7 kg/m^2^. The most prescribed medications in the OH group included alpha-adrenergic agonists, corticosteroids, and acetylcholinesterase inhibitors [2]. 11 patients were lost to follow-up or unreported after 7 years, leaving 47 OH patients (81% of initial sample) with complete follow-up.

HyperOH and OH cohorts were matched by age (70 ± 10 years vs. 70 ± 9 years, p > 0.9) and sex (56% male vs. 64% male, p = 0.5), but the BMI was greater in the OH cohort (24.3 ± 3.9 kg/m^2^ vs. 27.9 ± 4.7 kg/m^2^, p = 0.003) [1].

### Autonomic Assessment

The mean supine blood pressure was 149/79 ± 25/11 mmHg in the hyperOH cohort and 153/83 ± 27/13 mmHg in the OH cohort (SBP, p = 0.6, DBP, p = 0.1). The upright blood pressure in hyperOH was 115/70 ± 29/15 mmHg and 94/62 ± 17/11 mmHg in OH (SBP, p = 0.003; DBP, p = 0.02). Thus, the hyperOH cohort experienced a smaller orthostatic fall in blood pressure when compared to the OH cohort (34/9 ± 14/4 vs 59/21 ± 9/2, p = 0.05), despite experiencing a similar orthostatic increase in heart rate (9 ± 2 bpm vs 13 ± 3 bpm, p = 0.15). The ΔHR/ΔSBP ratio in the hyperOH group was 0.33 bpm/mmHg compared to the OH group with a ΔHR/ΔSBP ratio of 0.22 bpm/mmHg (p = 0.25) [2, 3]. Further, the hyperOH group had a mean Valsalva ratio of 1.2 ± 0.14, whereas the Valsalva ratio of the OH group was 1.1 ± 0.12 (p = 0.08) [1–3]. The mean PRT during Valsalva Maneuver for the hyperOH group was 16.5 ± 9 seconds compared to 32 ± 17 seconds in the OH group (p < 0.001) [1, 2]. See Table 1 for differences in demographics and clinical characteristics between hyperOH and OH.

The supine plasma norepinephrine level was 420 ± 179 pg/mL for the hyperOH patients and 116 ± 84 pg/mL for the OH patients (p < 0.0005). Upon standing, the plasma norepinephrine level increased to 938 ± 305 pg/mL when upright within the hyperOH group, whereas the OH cohort experienced a blunted orthostatic increase in plasma norepinephrine to 209 ± 159 pg/mL when upright (p < 0.0005), indicating the expected impairment of the sympathetic nervous system. Eighteen OH patients even displayed an orthostatic plasma norepinephrine level that remained below 100 pg/mL throughout the tilt test [2].

### Outcomes

The 7-year cumulative mortality rate was higher for hyperOH cohort compared to OH (35% vs. 22%, p = 0.01). Figure 2 shows the cumulative mortality rate for hyperOH and OH. The causes of death for deceased patients in the hyperOH cohort were MSA (n = 3) and malignancies (amyloidosis, n = 1, and multiple myeloma, n = 1), with several causes of death remaining unknown.

**Figure 1:**
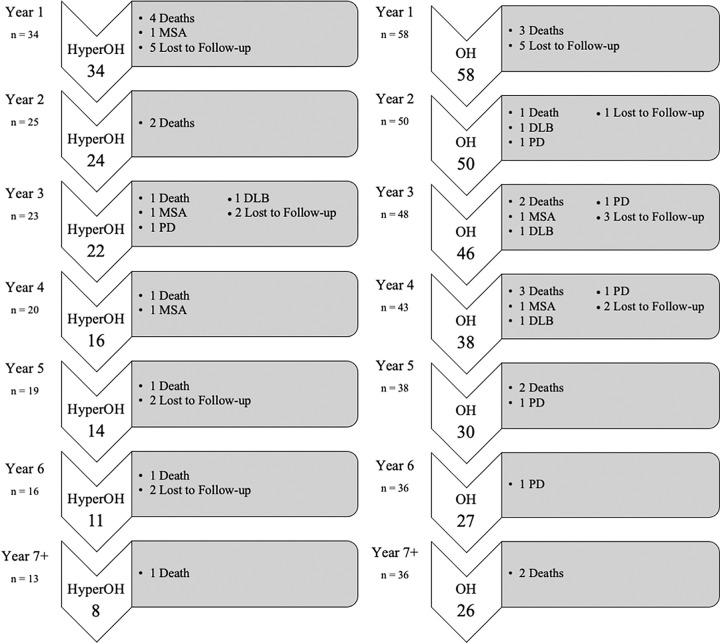
Outcomes of hyperOH (left) and OH (right) over 7 years including mortality, phenoconversion, and loss to follow-up

**Figure 2:**
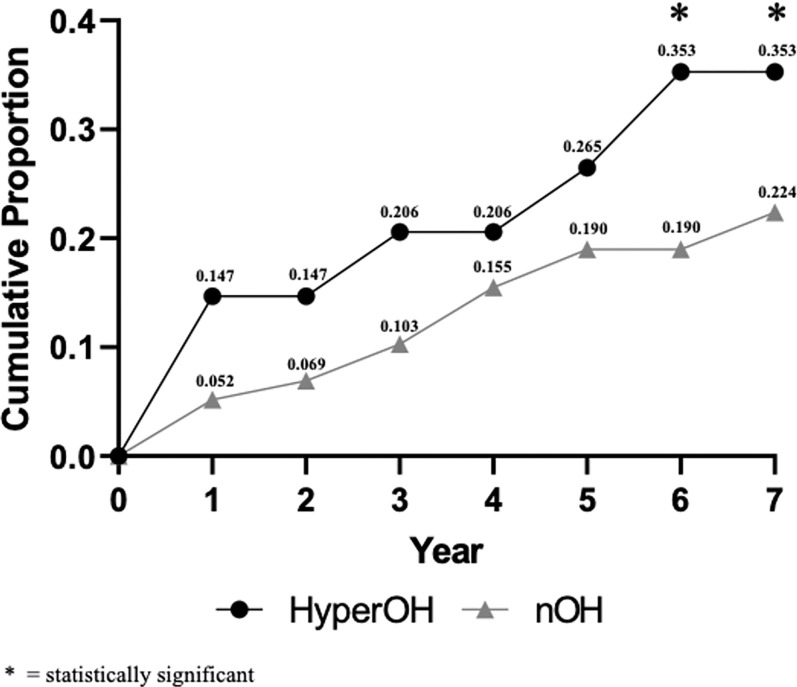
Cumulative mortality in hyperOH and OH over 7 years

**Figure 3:**
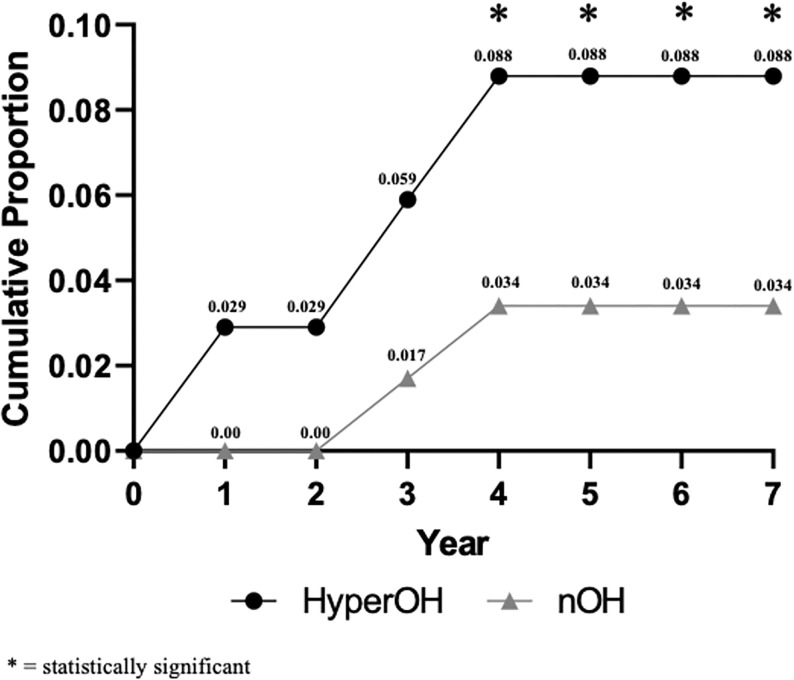
Cumulative phenoconversion to multiple system atrophy (MSA) in hyperOH and OH over 7 years

The hyperOH group had a 7-year cumulative MSA phenoconversion rate of 8.8%, compared to 3.4% in OH (p = 0.04). Within the hyperOH cohort, one patient converted to MSA during the first year after initial diagnosis, and two patients converted to MSA during years 3 and 4 after initial diagnosis. With regards to PD and DLB, OH patients had a non-significantly greater phenoconverstion rate compared to hyperOH over 7 years (p = 0.5 and p = 0.6, respectively). Figure 3 illustrates the cumulative proportion of patients who phenoconverted to MSA throughout the 7 years. Figure 1 depicts the number of patients who died, phenoconverted, or were lost to follow-up year-by-year over 7 years in hyperOH and OH.

## Discussion

Even though our autonomic testing confirmed that hyperOH patients have autonomic failure, these patients have a much greater increase in norepinephrine levels upon standing when compared to the OH cohort. This could indicate that postganglionic sympathetic nerves are partially preserved, suggesting early autonomic impairment or damage of the central autonomic commands, which occurs in MSA. Our autonomic testing also confirmed previous findings that hyperOH patients have less severe autonomic impairment than OH, as evidenced by a greater ΔHR/ΔSBP ratio and a shorter PRT during Valsalva Maneuver [1, 3].

Of note, the hyperOH cohort had a greater proportion of phenoconversion to MSA due to their normal or high norepinephrine levels that indicate pre-ganglionic lesions of the autonomic nervous system. On the other hand, the OH cohort with low norepinephrine levels converted more often to peripheral forms of autonomic failure (PD), supporting evidence for post-ganglionic lesions and sympathetic denervation.

HyperOH patients have poor prognosis given their higher mortality rate over the 7-year follow-up period. This suggests a possible partial denervation of the autonomic nervous system, which could signal an underlying illness with rapid progression that could eventually lead to complete autonomic failure. It is not surprising that there is a high prevalence of death for amyloidosis and MSA in the hyperOH cohort

The findings in this study highlights the prognostic value of norepinephrine in predicting the outcomes of patients with orthostatic hypotension. Considering the importance of norepinephrine as a pathophysiological biomarker in autonomic disorders, it would be important to incorporate upright norepinephrine in the clinical setting.

### Limitations

The main limitation to this study is the relatively small sample size of the cohort. Further, the loss of follow up throughout the study contributed to limiting the data that was available. Although procedures were standardized for all patients, catecholamine levels can be erroneously increased due to difficulties with intravenous catheter placements and blood draws.

## Conclusions

Although patients with hyperOH have less severe autonomic impairment than typical neurogenic OH patients, these data suggest that the long-term outcomes of hyperOH may be worse than those of OH, with a high mortality and MSA phenoconversion rate. Patients with hyperOH should be monitored carefully in the years following initial diagnosis.

## Figures and Tables

**Table 1:** Study population and clinical assessment of hyperOH and OH

Parameters	HyperOH	OH	Significance
Age (years)	70 ± 10	70 ± 9	p = 0.97
Sex (% male)	56%	64%	p = 0.46
BMI (kg/m^2^)	24.3 ± 3.9	27.9 ± 4.7	p = 0.03
SBP supine (mmHg)	149 ± 25	153 ± 27	p = 0.55
DBP supine (mmHg)	79 ± 11	83 ± 13	p = 0.11
HR supine (bpm)	73 ± 13	65 ± 11	p = 0.006*
NE supine (pg/mL)	420±179	116 ± 84	p< 0.0005***
SBP upright (mmHg)	115 ± 29	94 ± 17	p = 0.003**
DBP upright (mmHg)	70 ± 15	62 ± 11	p = 0.02*
HR upright (bpm)	82 ± 15	78 ± 13	p = 0.28
NE upright (pg/mL)	938 ± 305	209 ±159	p< 0.0005***
ΔSBP	34 ± 14	59 ± 9	p< 0.0005***
ΔDBP	9 ± 4	21 ± 2	p = 0.003**
ΔHR	9 ± 2	13 ± 3	p = 0.15
ΔHR/ΔSBP	0.33	0.22	p = 0.25
ΔNE	518 ± 126	94 ± 75	p< 0.0005***
VM Ratio	1.2 ± 0.14	1.1 ± 0.12	p = 0.08
PRT (seconds)	16.5 ± 9	32 ± 17	p< 0.001**

Values are means ± standard deviations. BMI = body mass index. SBP = systolic blood pressure. DBP = diastolic blood pressure. HR = heart rate. NE = norepinephrine. VM = Valsalva Maneuver. PRT = pressure recovery time.
